# Clinical characteristics and factors associated with survival rate of patients with non-muscle invasive bladder cancer attending at a Tertiary Hospital in Somalia

**DOI:** 10.1186/s12885-024-12632-9

**Published:** 2024-07-14

**Authors:** Abdikarim Hussein Mohamed, Khaled Ali Mohamed, Ertan Kayacan, Yassin Nur, Mohamed Abdikarim Nur-amin

**Affiliations:** 1 Mogadishu Somalia Turkish Training and Research Hospital, Mogadishu, Somalia; 2 University of Somalia, Mogadishu, Somalia

**Keywords:** Non-muscle invasive bladder cancer, Sub-saharan African countries, Intravesical chemotherapy, Mortality

## Abstract

**Background:**

A few studies regarding the epidemiology and risk factors of Non-muscle Invasive Bladder Cancer (NMIBC) are reported from Sub-Saharan African countries (SSA), including Somalia, and the African literature is scant on the management of NMIBC. The present study aims to evaluate the clinical-histopathological characteristics and factors associated with the survival rate of patients with NMIBC.

**Method:**

This six-year cohort study included 196 patients with NMIBC. It reviewed the clinical and histopathological characteristics and factors predicting cancer-specific survival for these patients.

**Results:**

The mean patient age was 59.01 ± 11.50 years, with a male-to-female ratio of 2.8:1. Urothelial carcinoma (UC) constituted the most common pathological type, accounting for 90.8%; Ta LG and T1HG were the most common histopathological tumour stage and grade (*n* = 90, 45.9%, vs. *n* = 56, 28.6%), respectively. The mean tumour size was 4.72 ± 2.81 cm. The cancer-specific mortality(CSM) was 13.3%. Age [2.252(2.310–2.943], *p* < 0.001], Gender [1.031(0.981-1.1.242),*p* < 0.001], tumour stage and grade [4.902(3.607–5.614),*p* < 0.001], tumour location [1.135(0.806–1.172),*p* < 0.001], number [0.510(0.410–0.920),*p* = 0.03], tumour size [1.523(0.936–1.541),*p* < 0.001], use of intravesical chemotherapy or BCG [2.810(1.972–4.381),*p* < 0.001], preoperative hydronephrosis grade [1.517(1.172–2.154),*p* < 0.001], and follow-up compliance [3.376(2.633–5.018),*p* < 0.001] were all associated with CSM. The 5-year overall survival was 57.1%, and cardiovascular diseases were the leading cause of mortality (*n* = 34), followed by diabetes (*n* = 28).

**Conclusion:**

Our study findings revealed that UC constituted the most common pathological subtype, though less than forty per cent of our patients receive intravesical adjuvant therapies, which are crucial to minimizing disease morbidity and mortality. Initiatives improving uro-oncological care, including subspecialty training in oncology and essential cancer therapies, better access to urology services, and cancer screening programs, are much needed for optimal management plans and care in the country.

## Introduction

Bladder cancer (BC) is the fourth and the 11th most common cancer in men and women, respectively [1]. The worldwide age-standardized incidence rate of BC is 9.5 in men and 2.4 in women per 100,000 person/year. On the other hand, the age-standardized mortality rate is 3.3 for men and 0.86 for women [1, 2]. A recent systemic review and meta-analysis conducted by Adeloye and colleagues reported that the pooled incidence of bladder cancer in Africa was 7.0 in men and 1.8 in women per 100,000 persons [3]. An estimated pooled incidence and mortality of 10.1 (7.9–11.9) and 2.0 (1.0–3.0) Vs. 5.0 (3.8–6.6) and 1.5 (0.9–2.0) per 100,000 persons/year among men and women were reported from North Africa and Sub-Saharan Africa (SSA), respectively [3].

75% of BC patients had a disease confined to the mucosa (stage Ta, carcinoma in situ) or tumor invading subepithelial connective tissue (stage T1) [2, 4]. This rate is even higher in younger patients under 40 [5].

The most significant risk factor for bladder cancer is tobacco smoking, which accounts for approximately two-thirds of all cases, followed by occupational exposures to carcinogens in developed countries. Contrarily, in Africa and the Middle East regions, Schistosomiasis infection is the common cause of bladder cancer [6, 7].

Disparities in risk factors, healthcare access to and delivery, and detection and diagnostic methods lead to global variations in incidence and mortality rates of bladder cancer [8].

A few studies regarding the prevalence and management of NMIBC cases were reported in the literature despite the increased incidence of bladder cancer in Africa over the last decades due to urbanization [3].

There have been no previous studies regarding NMIBC reported from Somalia. The present study aims to evaluate the clinical and histopathological characteristics and factors associated with the survival rate of patients with non-muscle invasive bladder cancer admitted at tertiary and sole oncology centers in Somalia.

## Method

A six-year cohort study was conducted to review the pathology results for patients who underwent transurethral resection of bladder tumors (TURBT) between January 2018 to December 2023. The data was retrieved using the hospital information system. Preoperative diagnosis of bladder cancer was based on the microscopic or gross hematuria, irritative voiding symptoms, or incidental finding of bladder tumors to investigate other medical conditions. Patients with histologically confirmed non-muscle invasive bladder urothelial carcinomas or non-urothelial variants were included in the study. Patients with regional involvement or distant metastases detected on radiological staging, previous history of muscle-invasive bladder cancer, incomplete or lack of histopathological data, and previous radiotherapy were excluded from the study.

The electronic medical records in the hospital information system (HIS) were retrospectively reviewed to assess the clinical characteristics, including patient age, gender, tobacco use and smoking, symptoms, and presence of preoperative hydronephrosis.

The study also assessed pathological characteristics such as tumor type, stage, tumor grade, and lymphovascular invasion. Clinical characteristics such as tumor size, location, number, and previous recurrence were also documented. Pathological T stage was reassigned according to the TNM Classification (2017, 8th edition). Tumor grade was evaluated according to the 2016 WHO classification by a single group of pathologists in our hospital. Lymphovascular invasion was defined as tumor cells in the lymphatic vessels and vascular walls.

Postoperative use of intravesical chemotherapy, bacillus Calmette-Guérin (BCG) or further surgery type was included in the analyzed variables. The five-year survival rate was evaluated, documenting the cancer-specific and all-cause mortality during the follow-up. The patient follow-up was reached through their appointment schedule and telephone from the hospital information system.

The approval form was received from the ethical research board committee of Mogadishu Somalia Turkish Training and Research Hospital (REF. MSTH-9006). In addition, informed consent was obtained from all patients. This study was carried out following the Helsinki Declaration contents.

All statistical analyses were used in the Statistical Package for Social Sciences (SPSS-IBM) for Windows version 26. Univariate descriptive statistics was used to analyze the variables. Multivariate analyses were performed to determine CSS and overall survival predictors. A hazard ratio (HR) with a 95% confidence interval (95% CI) was calculated. A p-value less than 0.05 was considered statistically significant.

## Results

This cohort included 196 patients with NMIBC who underwent TURBT during the study period. The patients’ histopathological tumor stage and grade were as follows: Ta LG accounted for nearly half of the cases (*n* = 90, 45.9%), followed by T1HG comprising 28.6% (*n* = 56) of the study population. T1 LG and Ta HG constituted 12.4% and 10.2%, respectively. UC constituted the most common pathological type, accounting for 90.8%. Approximately half of the patients were in the high-risk group (47%), followed by low-risk and intermediate-risk, accounting for 30.6% and 22.4%, respectively.

The mean patient age was 59.01 ± 11.50 years. The most frequent age group were patients between 60 and 79 years old (51%), followed by 40–59 years (22.4%), and elderly patients > 80 years (17.3%). No pediatric patients (i.e., < 18 years) were found in the records due to the hospital’s lack of a pediatric rectoscope. More than two-thirds of the study population were males (73.5%), with a male-to-female ratio of 2.8:1. About half of the patients had microscopic hematuria, while gross hematuria and incidental findings were the following clinical manifestations. Nearly half of the cases (49%) were smokers. Thirty-one per cent of cases had some degree of hydronephrosis. Detailed sociodemographic and clinical manifestations are displayed in Table 1.

**Table 1:** Sociodemographic and clinical manifestation of the patients

Variable	No. of patients/Percentage
**Age**	
Mean age 19–39 40–59 60–79 > 80	59.01 ± 11.50 years 18 (9.2%) 44 (22.4%) 100 (51%) 34 (17.3%)
**Gender**	
Male Female	144 (73.5%) 52 (26.5%)
**Symptoms**	
Microscopic hematuria Gross hematuria Irritative voiding symptoms Incidental	112 (57.1%) 36 (18.4%) 18 (9.2%) 30 (15.3%)
**Tobacco Use and Smoking**	
Yes No	96 (49%) 100 (51%)
**Type**	
UC SCC Others	178 (90.8%) 12 (6.1%) 6 (3.1%)
**Tumor stage and grade**	
Ta LG Ta HG T1 LG T1 HG CIS	90 (45.9%) 20 (10.2%) 24 (12.4%) 56 (28.6%) 6 (3.1%)
**Risk groups**	
Low Intermediate High risk	60 (30.6%) 44 (22.4%) 92 (47%)
**Location**	
Posterolateral Anterior Bladder neck	156 (79.6%) 26 (13.3%) 14 (7.1%)
**Size**	
Mean < 3 cm > 3 cm	4.72 ± 2.81 cm 62 (31.6%) 134 (68.4%)
**Number**	
Single Multiple	102 (52%) 94 (48%)
**Primary tumor**	
Primary Recurrent	168 (85.7%) 28 (14.3%)
**LVI**	
Yes No	24 (12.2%) 172 (87.8)
**Hydronephrosis grade**	
No G1 G2 G3 G4	134 (68.4%) 24 (12.2%) 18 (9.2%) 8 (4.1%) 12 (6.1%)
**Immediate single installation**	
Yes No	74 (37.8%) 122 (62.2%)
**Induction therapy**	
BCG Mitomycin C	44 (22.4%) 12 (6.1%)
**Maintenance therapy**	
BCG	16 (9.2%)
**5-year cancer-specific death**	
Yes No	26 (13.3%) 170 (86.7%)
**5-year overall death**	
Yes No	84 (42.9%) 112 (57.1%)

The mean tumour size was 4.72 ± 2.81 cm. In the multivariate analyses, Age [2.252 (2.310–2.943], *p* < 0.001], Gender [1.031 (0.981-1.1.242), *p* < 0.001], tumour stage and grade [4.902 (3.607–5.614), *p* < 0.001], tumour location [1.135 (0.806–1.172), *p* < 0.001], number [0.510 (0.410–0.920), *p* = 0.03], tumour size [1.523 (0.936–1.541), *p* < 0.001], use of intravesical chemotherapy or BCG [2.810 (1.972–4.381), *p* < 0.001], preoperative hydronephrosis grade [1.517 (1.172–2.154), *p* < 0.001], and follow-up compliance [3.376 (2.633–5.018), *p* < 0.001] were all associated with CSM (Table 2).

**Table 2:** Multivariable analysis for factors predicting cancer-specific survival

Variable	HR	95% CI	*p*-value
Age	2.252	2.310–2.943	0.001
Gender	1.031	0.981-1.1.242	0.001
Tumor stage and grade	4.902	3.607–5.614	0.001
Location	1.135	0.806–1.172	0.001
Number	0.510	0.410–0.920	0.03
Size	1.523	0.936–1.541	0.001
Use of intravesical chemotherapy or BCG	2.810	1.972–4.381	0.001
Preoperative Hydronephrosis grade	1.517	1.172–2.154	0.001
Follow-up compliance	3.376	2.633–5.018	0.001

Regarding intravesical installations, 37.8% of the cases get immediate single instillation, mainly mitomycin C installation. For intermediate and high-risk groups, 22.8% received BGC induction, 6.6% for Mitomycin C induction, and only 9.2% completed a maintenance BCG therapy, while six patients underwent a radical cystectomy. All patients who needed BCG or radical cystectomy were sent abroad for the unavailability of the whole country.

In terms of regular follow-up schedules, 42% of the patients maintain the follow-up schedules per EAU guidelines for the first year, while this percentage significantly reduced in 18% of the cases for the second year due to inability to access healthcare services, poor socioeconomic status, insufficient tertiary hospitals in the country and rural distribution of the patients.

The time period of the patients included in the study was six months to 72 months. The cancer-specific mortality was 13.3% (*n* = 26, 17 male and nine female), 14 with T1HG, 6,4,2 with T1LG, high-grade CIS, and TaHG, respectively. Sixteen patients with non-urothelial variants and ten UC were among those who died. Advanced age, tumour size > 3 cm, multiple, recurrent tumors, and tumors located in the bladder neck were significantly associated with CSM. The 5-year overall survival was 57.1%, and cardiovascular diseases were the leading cause of mortality (*n* = 34), followed by diabetes (*n* = 28) and bladder cancer (*n* = 26).

Three cases had upper urinary tract urothelial cancer, while three other cases had schistosomiasis-induced SCC of the bladder.

## Discussion

A few studies regarding the epidemiology and risk factors of NMIBC are reported from Sub-Saharan African countries (SSA), including Somalia, and the African literature is scant on the management of NMIBC. A systematic review on the current trend of non-muscle invasive bladder cancer in Africa conducted by Cassell and associates examined a total of 12 studies carried out across 9 African countries (Egypt, Nigeria, Senegal, Benin, Mali, Zambia, Lybia, Ethiopia, and Tanzania) between 2000 and 2019 [9]. Most of these studies were retrospective hospital-based studies lacking structured bladder cancer guidelines. To date, there have been no previous studies regarding bladder cancer reported from Somalia, and there is no national cancer registry database. The present study aims to evaluate the epidemiological, histopathological characteristics and factors associated with the survival rate of patients with non-muscle invasive bladder cancer admitted at tertiary and sole oncology center in Somalia.

In our study, the most frequent age group were patients between 60 and 79, comprising half of the cases, followed by 40–59 years. In contrast to our findings, A systematic review and Bayesian network meta-analysis regarding the epidemiology of bladder cancer in Africa conducted by Adeloye and associates included a total of 22 studies carried out across 15 African countries: eight studies from North Africa, one from Central Africa, four from East Africa, four from Southern Africa and five from West Africa between January 1980 through June 2017, reported a pooled that participants aged 50 − 59 years, followed by 40–49 years age group (30%) with a mean age ranging 36.8 to 61 years [3]. The discrepancies are partially due to geographic and ethnic variations as well as the etiological risk factors.

Our results show a significantly higher rate of bladder cancer among men compared with women, which corresponds to the previously published studies [10]. A global overview of recent patterns of bladder cancer incidence and mortality demonstrated that 76.87% of incidence and 74.54% of mortality from bladder cancer were found to occur in males, showing that men are 4–5 times more likely to be diagnosed with bladder cancer than women, which is in line with our study findings [11]. A higher rate of smoking and increased occupational exposure leads to this greater incidence and mortality from bladder cancer in men.

The presentation of bladder cancer in Africa differs from that observed in developed countries. Urothelial carcinoma (UC) is the most predominant histological subtype of bladder cancer in developed countries. Meanwhile, squamous cell carcinoma (SCC) is the most common type in most parts of Africa. A meta-analysis using the PRISMA guidelines included 23 hospital-based articles concerning bladder cancer in Africa reported that SCC is still the most predominant subtype in most African countries, constituting about 53–69%, though the recent studies noted a notable increase in UC in Africa representing 9–41% of the cases [12]. The epidemiology of cancer of the bladder key predominant subtypes in nine African countries was as follows: UC was the primary subtype in Ethiopia (80%), Kenya (53-67%), and South Africa (95%), while SCC was the primary subtype in Zambia (71%-60%), Egypt (53%), Zimbabwe (52-71%), Nigeria (39-66%), Senegal (58%), and Tanzania (18-72%) [12]. In our study, UC constituted the most common pathological type, accounting for 90.8%. Deuker and colleagues conducted a comparative study evaluating the stage at presentation, treatment rates, and cancer-specific mortality (CSM) of non-urothelial variant histology (VH) bladder cancer (BCa) relative to urothelial carcinoma of the urinary bladder (UCUB), included 222,435 patients with BCa [13]. The rate of VH was 5%, squamous cell carcinoma 1.6%, adenocarcinoma 0.8%, neuroendocrine carcinoma 0.8%, and other VH BCa 1.7%. A higher rate of VH of 9.2% was noted in our study.

Ta accounts for most NMIBC (60%), whereas T1 and Tis (CIS) account for 30% and 10%, respectively [9]. In contrast, our study findings reported that Ta accounts for 56.1% of the total cases, whereas T1 represented 41% and CIS 3.1%. A descriptive histopathological pattern of bladder cancer in Nigeria reported that 40.6% of cases had a disease confined to the mucosa (PTa), while 29.7% had a lamina propria invasion (PT1) [10]. A hospital-based retrospective cross-sectional analysis reviewing the pattern and surgical management of bladder tumors from Ethiopia reported that 85.5% of bladder tumors were non-muscle invasive, of which 74.7% of patients had a low-grade, and 23.0% had a high-grade disease [14].

There are notable differences in the proportion of UC and SCC across individual African countries due to schistosomiasis infection, which is largely associated with SCC, smoking, industrialisation, and Westernized lifestyles, which are largely associated with UC. Urinary schistosomiasis is an endemic disease in many low and middle-income countries, and schistosomiasis-associated bladder cancer usually presents in more advanced stages. However, when adjusting for stage, risk status, and comorbidities, the oncologic outcomes are generally comparable for schistosomiasis-associated bladder and non-schistosomiasis-associated bladder [15, 16]. As observed in our cohort, approximately 50% of BC cases are caused by tobacco smoking, increasing the risk by three to four times [2].

Intravesical adjuvant chemotherapy and immunotherapy are crucial to minimize the likelihood of recurrence rate. However, Radical cystectomy performed early is the gold standard for treating high-grade tumors with variable histology, as well as multiple and recurrent tumors. For patients presumed to be at low risk, one immediate chemotherapy installation is indicated. Patients with an intermediate risk should receive one year of full-dose BCG or chemotherapy instillations for a maximum of 1 year, while for those with high-risk tumors, full-dose intravesical BCG for 1–3 years is recommended [17]. Although intravesical therapies reduce disease recurrence and progression, unfortunately, less than forty per cent of our patients receive these therapies. There are very few specialists who can manage and perform TURBT, and there is a lack of neoadjuvant and adjuvant chemotherapy in the country.

Various potential mechanisms contributing to the recurrence of NMIBC have been documented [18]. These include the presence of undetected cancer during cystoscopy, the persistence of disease after transurethral resection due to incomplete removal of the tumour bed, re-implantation of cancer cells during resection, drop metastasis from the initial upper tract cancer, and the field change cancerization effect. While the progression of NMIBC to muscle-invasive bladder cancer (MIBC) is common, ranging from 10 to 30% at five years, dysregulation of the immune microenvironment (i.e., tumour-infiltrating immune cells (TIICs) and immune-related genes) promotes the progression [19]. Data regarding recurrence, progression, and metastatic rates were not reported in our study since most of our cases did not present the follow-up appointment schedule; only 18% were on the follow-up program in the second year and lost later. The main reasons were the patients’ socioeconomic status, rural distribution, and low educational level.

Patients with NMIBC need surveillance following therapy. Nearly two-thirds of our patients become lost to follow-up and could present later with an advanced stage of the disease. A low rate of public awareness of cancer, underfinanced and fractured health systems, inaccessible cancer screening services supporting early diagnosis and prompt treatment, and a lack of subspecialty training in oncology and essential cancer therapies result in suboptimal management plans and care in Africa [20].

Our patients’ 5-year CSS and OS were 86.7% and 57.1%, respectively. An overview study for 220,405 patients diagnosed with UBC from 1993 to 2012 extracted from the Surveillance, Epidemiology and End Result (SEER) 18 database, investigating the mortality and survival outcomes reported a 0.709, 0.609 and 0.421, and 0.861, 0.831 and 0.789 of 3-, 5- and 10-year OS and CSS rates. In line with our study findings, the authors stated that BC patients are 44.5 times more likely to die of CVD when compared with the general population, and cardiovascular health should be underscored to lower mortality [21]. Bladder cancer is approximately three times more prevalent in men than women. Nevertheless, women have a poorer prognosis. In our study, women had a 17.3% mortality rate compared to men with 11.8%. Corresponding to our study, A large review study for the Swedish Urinary Bladder Cancer Register between 1997 and 2014 regarding Sex Differences in Urothelial Bladder Cancer Survival conducted by Cecilia and associates reported that women had a higher bladder cancer mortality (adjusted hazard ratio, 1.15; 95% confidence interval, 1.08–1.23) among those diagnosed with bladder cancer [22].

This paper examines the current state of urological care in Somalia, focusing on several critical issues prevalent across Africa. These include low public awareness of cancer, inadequate follow-up rates for treatment, underfinanced and fragmented health systems, limited access to cancer screening hindering early diagnosis and timely treatment, and a scarcity of specialized training in oncology and urology.

This study has several limitations, including its retrospective single-centre study with a small sample size. Second, due to the patient’s socioeconomic status and rural distribution, most patients are reached through a telephone and their educational background could be low, which has a negative impact on oncological outcomes. Third, the analysis of intrinsic subtypes and phenotypes of high-grade bladder cancer was not available throughout the country. Despite these limitations, this is the first study investigating the clinical and histopathological characteristics and factors associated with the survival rate of patients with NMIBC.

## Conclusion

Our study findings revealed that UC constituted the most common pathological subtype, and non-invasive low-grade urothelial carcinoma was the most common histopathological tumor stage and grade. Also, our study reported that less than forty per cent of our patients receive intravesical adjuvant chemotherapy and immunotherapy, which are crucial to minimizing disease morbidity and mortality. Initiatives improving uro-oncological care, including subspecialty training in oncology and essential cancer therapies, better access to urology services, and cancer screening programs, are much needed for optimal management plans and care in the country.

## Data Availability

All study data and materials can be obtained from the corresponding author.

## Abbreviations

BC: bladder cancer
BCG: bacillus Calmette-Guérin
CSM: cancer-specific mortality
HIS: hospital information system
LVI: lymphovascular invasion
NMIBC: Non-muscle Invasive Bladder Cancer
SSA: Sub-Saharan African countries
SCC: squamous cell carcinoma
TURBT: transurethral resection of bladder tumors
UC: urothelial carcinoma
